# A Novel COVID Era-Related Oromandibular Dyskinesia: Surgical Mask-Induced Dyskinesia?

**DOI:** 10.1017/cjn.2021.139

**Published:** 2021-06-21

**Authors:** Fahimeh H. Akhoundi, Anthony E. Lang, Saeed Ghazvinian, Ahmad Chitsaz, Maziar Emamikhah, Mohammad Rohani

**Affiliations:** Division of Neurology, Firoozgar Hospital, Iran University of Medical Sciences, Tehran, Iran; Edmond J Safra Program in Parkinson’s Disease and the Morton and Gloria Shulman Movement Disorders Clinic, Toronto Western Hospital, UHN, Division of Neurology, University of Toronto, Toronto, Ontario, Canada; Division of Neurology, Atieh Hospital, Tehran, Iran; Department of Neurology, School of Medicine, Isfahan Neuroscience Research Center, Isfahan University of Medical Sciences, Isfahan, Iran; Division of Neurology, Hazrat Rasool Hospital, Iran University of Medical Sciences, Tehran, Iran; Skull Base Research Center, Five Senses Health Institute, Iran University of Medical Sciences, Tehran, Iran

**Keywords:** Orolingual dyskinesia, Oromandibular dystonia/dyskinesia, Respiratory protective devices, Surgical mask, COVID-19

Throughout the COVID-19 pandemic, wearing facemasks has become mandatory in many parts of the world. While wearing a mask has a vital role in protecting people against the spread of infection, it has not been free of complications. We are reporting two unique cases of oromandibular dyskinesia induced by wearing a surgical mask.

A 58-year-old right-handed man was brought to our attention because of abnormal movements in the oromandibular area (Case 1). His wife reported that the abnormal movements started 3 months earlier, when he started wearing a surgical mask due to the COVID-19 pandemic. The patient denied any inner urge to perform the movements or abnormal sensation over this area; he was unaware of the movements and was not disturbed by them as the movements did not interfere with his daily activities such as speaking and eating. He reported a 1-year history of hand tremor present at rest, for which he had tried primidone, clonazepam, and propranolol without benefit but had not received anti-Parkinson medications. He had no history of dopamine receptor blocking drugs usage, tics, or obsessive compulsive disorder. He had a normal dentition and no history of oral or facial sensory complaints.

On neurologic examination, he had normal mental state and cognition (MoCA score was 30 out of 30). He had mild hypokinesia and tremor of the left limbs present at rest. There might have been very occasional and inconsistent low-amplitude side-to-side jaw movements in the absence of wearing the mask; distracting/activating tasks did not bring out any abnormal movements, nor did voluntarily activating facial muscles, talking or protruding the tongue (video 1). However, wearing a surgical mask elicited abnormal movements in the form of oromandibular dyskinesia which was further enhanced by distraction (video 2). When he took away the mask, the abnormal movements completely disappeared. We asked him to wear a different kind of mask (an N95 mask) and we observed an almost complete resolution of the dyskinesia (video 3). His neurologic examination was otherwise entirely normal, including normal cranial nerves, and normal motor and sensory examination.

His brain MRI displayed white matter T2 hyperintensities compatible with severe diffuse small vessel disease. We started low dose of levodopa-benserazide, with some improvement of both the Parkinsonism and the oromandibular dyskinesia.

The second case, a 38-year-old man presented to us with abnormal facial contractions, which were only present when he was wearing a surgical mask since the beginning of COVID-19 pandemic. Initially, these movements were mild but gradually they became more severe and bothersome. According to the patient, there was no inner urge or sensations to induce these movements and they were completely insuppressible.

He was an IT engineer with the unremarkable previous medical and familial histories. His drug history was also negative, especially for any neurotropic medications.

Neurologic examination without mask was completely normal. On wearing a surgical mask, there was abnormal contractions in lower face with more prominent involvement of the oromandibular area causing side-to-side deviation of the jaw.

Removing the surgical mask resulted in complete disappearance of the abnormal movements (video 4).

Brain MRI and laboratory tests including evaluation for Wilson’s disease were entirely normal.

The COVID-19 pandemic has deeply impacted the lives of the world’s population; one of them being the need to use respiratory protective devices including facemasks in public spaces. Wearing masks has been associated with a number of complaints, including fatigue, headache, impaired concentration, and drowsiness.^1,2^ We report two unique cases of oromandibular dystonia/dyskinesia (OMD) induced by wearing a surgical mask.

Whether these movements should be considered similar to a task-specific dystonia is uncertain. Task-specific dystonia is defined as a focal isolated dystonia that presents as loss of motor control occurring almost exclusively during a skilled movement or specific action.^3^ Although wearing a facemask is not a skilled movement, it does result in some tension and contraction of facial muscles.

Sensory tricks, also called geste antagoniste, represent the temporary alleviation of dystonia by a specific maneuver; sometimes even thinking or preparing to perform the specific maneuver results in amelioration of the dystonia, pointing to a disturbance of sensory motor integration in the pathophysiology of dystonia.^4^ Patients with oromandibular dystonia usually use sensory tricks such as chewing gum or holding something in their mouth in order to improve their symptoms.^5^ Wearing a mask might hypothetically serve as a sensory trick for patients with oromandibular dystonia and result in the alleviation of their symptoms. However, in these cases, we see the opposite effect of the facemask as the inducer of the abnormal movement. In Case 1, this seemed to be selective for a softer facemask than a firmer more molded N95 mask, possibly due to the greater sensory input from more contact with the skin of the face in the former.

One point against calling this movement disorder dystonia is the lack of awareness of its presence in the first patient. This anosognosia-like feature is not at all characteristic of dystonia but is more typical of some choreic disorders such as levodopa-induced dyskinesia and chorea in Huntington’s disease.^6^ In the orolingual region, tardive orobuccolingual dyskinesia and edentulous dyskinesia are important examples of this phenomenon.^7^ The ameliorative effect of wearing dentures in both of these disorders^8^ is an interesting contrast to the exacerbating effect of wearing the surgical mask in our patients.

The first patient had some additional Parkinsonism and evidence of diffuse small vessel disease on brain MRI. Whether his abnormal movements on wearing a mask could be attributed to these findings is unclear. However, the second patient had a completely normal neurologic examination without the mask on, and a normal brain MRI which stand against this possibility.

To conclude, we report two patients with oromandibular dyskinesia exclusively induced by wearing a surgical mask. To our knowledge, this is the first report of abnormal movement induced by respiratory protective devices.

## Supplementary material

For supplementary material accompanying this paper visit http://dx.doi.org/10.1017/cjn.2021.139. click here to view supplementary material
