# Supplementary material for: A Novel COVID Era-Related Oromandibular Dyskinesia: Surgical Mask-Induced Dyskinesia?
Source: Can J Neurol Sci. 2021 Jun 21:1–2. doi: 10.1017/cjn.2021.139 (PMC8314084; doi:10.1017/cjn.2021.139)
Supplement: Supplementary file 1 [file S0317167121001396sup001.zip › S0317167121001396sup005.docx]

Video legends.

Video 1. This video shows mild hypokinesia on limbs. Throughout the examination there is no oromandibular dyskinesia.

Video 2. This video reveals very mild OMD without mask but significant side to side jaw movements with wearing the mask.

Video 3. On this video wearing a different mask (N95) abolishes completely the jaw movements.

Video 4. Second patient with facial dyskinesia on wearing a surgical mask and complete resolution of symptoms when he removes the mask.
